# A Rose by Any Other Name: The Long Intricate History of Localized Aggressive Periodontitis

**DOI:** 10.3390/pathogens13100849

**Published:** 2024-09-29

**Authors:** Daniel H. Fine, Helen Schreiner, Scott R. Diehl

**Affiliations:** Department of Oral Biology, Rutgers School of Dental Medicine, 110 Bergen Street, Newark, NJ 07101, USA; hschrein@sdm.rutgers.edu (H.S.); diehlsd@sdm.rutgers.edu (S.R.D.)

**Keywords:** aggressive periodontitis, *Aggregatibacter actinomycetemcomitans*, treatment success, consensus conferences, microbiome consortia, damage/response framework

## Abstract

This review addresses the recent World Workshop Consensus Conference (WWCC) decision to eliminate Localized Aggressive Periodontitis (LAgP) in young adults as a distinct form of periodontitis. A “Consensus” implies widespread, if not unanimous, agreement among participants. However, a significant number of attendees were opposed to the elimination of the LAgP classification. The substantial evidence supporting a unique diagnosis for LAgP includes the (1) incisor/molar pattern of disease, (2) young age of onset, (3) rapid progression of attachment and bone loss, (4) familial aggregation across multiple generations, and (5) defined consortium of microbiological risk factors including *Aggregatibacter actinomycetemcomitans*. Distinctive clinical signs and symptoms of LAgP are presented, and the microbial subgingival consortia that precede the onset of signs and symptoms are described. Using Bradford–Hill guidelines to assess causation, well-defined longitudinal studies support the unique microbial consortia, including *A. actinomycetemcomitans* as causative for LAgP. To determine the effects of the WWCC elimination of LAgP on research, we searched three publication databases and discovered a clear decrease in the number of new publications addressing LAgP since the new WWCC classification. The negative effects of the WWCC guidelines on both diagnosis and treatment success are presented. For example, due to the localized nature of LAgP, the practice of averaging mean pocket depth reduction or attachment gain across all teeth masks major changes in disease recovery at high-risk tooth sites. Reinstating LAgP as a distinct disease entity is proposed, and an alternative or additional way of measuring treatment success is recommended based on an assessment of the extension of the time to relapse of subgingival re-infection. The consequences of the translocation of oral microbes to distant anatomical sites due to ignoring relapse frequency are also discussed. Additional questions and future directions are also presented.

## 1. Introduction: Importance of Recognizing Subtypes of Periodontitis for Scientific Discovery

The primary purpose of this hybrid review is to present evidence that Localized Aggressive Periodontitis (LAgP; recently reclassified as Stage III Grade C Periodontitis) is a unique disease that deserves its own classification as a distinct form of periodontitis. The aim of this review is to describe the features that make this disease distinctive on a clinical and microbiological level. A more complete understanding of the roles of genetics and host responsiveness in young individuals requires additional research (described in Miguel and Shaddox (This Special Issue). Additional efforts to gain this important information may be thwarted if LAgP is abandoned as a distinct entity. This review will be structured into sections that support the role of *A. actinomycetemcomitans* in this unique disease. The manuscript is somewhat unconventional and consists of a hybrid paper that contains literature review material, opinions based on evidence, and examples of LAgP related to tooth loss and assessment of treatment success or failure. The paper stresses the clinical and microbiological aspects of LAgP, especially in vulnerable populations. The review presents these unique features in distinctive sub-headings as follows: (1) Overall Introduction, (2) Influence of the World Workshop Consensus Conference (WWCC) on Disease Classification, (3) Benefits of Precise Disease Classification, (4) Brief History of Landmark Experiments that Support Microbiological Associations with Dental Diseases, (5) The Damage/Response Framework, (6) Examples of Distinct Characteristics of LAgP, and (7) Conclusions and Future Directions.

The paper presents information that challenges the decision made by the WWCC to eliminate LAgP as a unique disease entity [1,2]. Many ways of classifying different forms of periodontal disease have been proposed, but the recent Stage III Grade C classification no longer recognizes the disease previously known as Localized Aggressive Periodontitis (LAgP), Periodontosis, Localized Juvenile Periodontitis, and Early Onset Periodontitis [1,3]. The Staging and Grading method of classification adopted by the leadership of the WWCC is a scheme that has been used extensively by cancer researchers and clinicians for purposes of creating a consistent system for prognosis and treatment [4]. However, the classification of the disease by staging and grading was never intended to serve as a substitute for a robust framework for disease diagnosis [5]. As noted above, throughout the extensive history of efforts to characterize LAgP, many name changes have evolved, but several features unique to this disease have remained constant, including (1) the early age of onset of disease, (2) the disease is localized, at least initially affecting incisors and first molars, and (3) the rapid rate of severe bone loss compared to forms of periodontitis presenting in adults [3,6]. It has also been obvious that, unlike adult periodontitis, LAgP frequently exhibits strong familial aggregation with transmission across multiple generations [7]. LAgP also exhibits a far higher prevalence of 2.05% in African Americans aged 14–17 compared to only 0.14% in persons of European ancestry in the United States, a nearly 15-fold difference in frequency [8].

The revised classification of LAgP as Stage III Grade C ignores the distinct clinical, microbiological, and genetic differences in this aggressive form of periodontitis as compared to adult forms of the disease [9]. Historical revisions of nomenclature that reflect shifting opinions are valid, but in this case, the WWCC change fails to reflect the most current scientific knowledge, especially with respect to clinical and microbiological features [10]. One goal of this review is to illustrate the negative effects that this “new” classification is already having on research aimed at understanding the etiology and progression of LAgP and the impacts that this “classification” has on clinical diagnosis and treatment. This paper will focus on (1) the unique clinical presentation of LAgP, (2) the unique microbiological risk factors associated with LAgP, and (3) historical and clinical examples that show why LAgP should continue to be recognized as a unique disease entity.

## 2. Influence of Consensus Conference and Key Microbiological Influences on Disease Classification

(A) WWCC impact on Disease Classification.

Dental biology does not exist in a vacuum. The WWCC focus on histopathological differences as the ultimate discriminator for periodontal disease classification is narrow. Histopathological discrimination is unlikely until biopsy material related to active disease has been collected [11]. In time, it is likely that both histopathological and genetic differences between periodontitis in older adults and LAgP in children and young adults will be uncovered [12]. However, at this moment, it is inappropriate to ignore the major clinical signs and symptoms that are unique to LAgP [13]. As for genetics, thus far, etiology based on a single gene of major effect causing LAgP appears unlikely, aside from rare syndromic forms such as those caused by mutations in the cathepsin C gene [14]. Instead, it is more probable that an ensemble of genes of small to moderate effect, combined with environmental risk factors, play a prominent role in susceptibility to the disorder [12,15]. The WWCC argument that cases of LAgP are too rare to warrant recognition as a unique diagnosis is inconsistent with standard practice in medicine where orphan diseases (affecting less than 200,000 individuals in the U.S.) routinely receive specific diagnoses [16]. The 2.05% prevalence among the 11+ million African American adolescents in the United States alone would mean that approximately 225,000 individuals are likely to be affected by LAgP. Moreover, necrotizing diseases are also very uncommon, but despite their limited numbers, these diseases were still recognized with a unique diagnosis by the WWCC [17,18]. The suggestion that the determinations of the WWCC are unalterable is invalid because changes in the WWC classification have occurred previously [19].

Necrotizing diseases such as Acute Necrotizing (AN) Gingivitis and AN Periodontitis provide good examples as to how disease categorization can be implemented by the WWCC based on new information despite their low prevalence. Further, AN diseases illustrate how “impairment of the host immune system” can create a dysbiotic state. In the examples of necrotizing diseases, histopathological differences are extolled [17]. We propose that patients with LAgP fit into the same category, also having an impaired host immune system but in this case at the local level, as demonstrated by polymorphonuclear leukocyte dysfunction that in itself supports the concept that this is a specific category of disease [20]. In spite of these inconsistencies, the WWCC authors failed to create a separate case definition for LAgP as a distinctive disease entity [1]. There is no recognition of the uniqueness of LAgP in the WWCC decision despite the fact that many expert reports in the literature, including the foundation paper on LAgP written for the WWCC, clearly point to specific signs and symptoms, especially in patients of African descent who have excessive bone loss at an early age [11,21]. In fact, the decision to eliminate LAgP as a disease entity, despite its characteristically unique clinical features that separate it from other forms of periodontitis, lacked the widespread agreement among the Workshop participants normally required for a decision to be characterized as a “consensus” [22].

(B) Support for Maintaining LAgP as a Disease entity.

We have used this section to provide support for maintaining LAgP as a distinct disease entity, offering a rationale for this support that is more accessible to both clinicians and researchers.

Over the years, disease diagnoses have been debated among the “lumpers” and “splitters”. These terms originated in evolutionary biology but could be used in any form of biology [22]. Most simply put, “lumpers” tend to join things together, while ‘splitters” separate things so that they can be examined individually in greater detail. In the words of Siddhartha Mukherjee, author of the bestselling book “*The Emperor of All Maladies*” [23], there is great value in differentiating cancer types for diagnosis and therapy. In his words, “How can you treat something you can’t name?”. As one example, Dr. Mukherjee points out that targeted immunotherapy is successful because definitions of disease highlighting different types of cancers can be affected by specific targeted treatments [23]. This reasoning can also be applied to LAgP. Staging and Grading, first introduced in the 1940s and 1950s by Dr. Pierre Denoix, was never intended as a diagnostic strategy [24].

Personalized/Precision Medicine was promulgated by the insightful work of Dr. Ralph Snyderman (former member of the immunology section of the National Institute of Dental and Craniofacial Research and Dean of Duke University Medical School) [25]. Personalized Medicine makes great efforts to define diseases precisely as opposed to lumping different forms of a disease into a single group [21].

i. Age as a consideration for case determination. A close look at the new WWCC definition of LAgP (now called Stage III Grade C Periodontitis) reveals that there is no distinction within this framework that discriminates between a young adolescent with the disease and older patients presenting with a similar pattern of disease [13]. It appears as if the extensive data on cases of Localized Aggressive Periodontitis in adolescents or children who show extreme levels of disease were overlooked for reasons not enumerated [26].

There is no dispute that subgingival bacteria initiate a host damage response in both LAgP and other forms of periodontitis [27]. However, it appears as if factors such as age of onset, ethnicity, and rate of disease progression are ignored in the recent Consensus document [18]. The Consensus summary states that differences in the pathobiology of aggressive and chronic forms of periodontitis are not significant enough to establish distinct disease entities [1]. Of curiosity, age as a factor for periodontitis is reported by one of the foundation papers, but here, age assessment begins with individuals 30 and above [28]. The paper concludes that …”empirical evidence-driven definitions of CAL (Clinical Attachment Level) thresholds signifying disproportionate severity of periodontitis by age are feasible [28] and that …”age is a significant determinant of the clinical presentation of periodontitis…” and “… individuals with fewer remaining teeth have higher mean CAL and PD measures” [28]. The age determinant is especially true for LAgP, but the age range for an LAgP disease classification is not considered in the paper. The focus of those of us who study LAgP has usually been on individuals aged 20 and younger, but this age group was not recognized [29].

ii. Rate of bone loss as a consideration for disease classification. The most prominent illustration of differences in the rate of disease progression occurs when comparing LAgP to Adult Periodontitis (AP). The rate of disease progression is a difficult parameter to measure unless longitudinal studies are performed [30,31]. A simple examination of specific site disease progression can be determined when LAgP and adult periodontitis patients are compared and age is introduced as a parameter. There is no denying that 6 to 8 mm of bone loss is rarely seen in a short period of 2–3 years in any other forms of periodontitis aside from LAgP [11,32]. However, even in LAgP, this very significant fact (rate of bone loss) is hidden when mean clinical attachment levels (CAL) are averaged over all teeth with up to 168 sites [30] (see Section 6 B).

iii. Etiology as a consideration for disease classification. The hazardous, often challenging, shifting, and, at times, impaired oral environment (e.g., irradiated salivary glands, dry mouth, circadian rhythms) has resulted in a highly adaptable oral microbiome [33]. As the main entryway into the body, bacteria first reaching the oral cavity face enormous environmental obstacles and a multitude of surfaces for colonization [34]. The more we explore, the more we realize that oral bacteria are not only involved in dental disease but that these highly adaptable bacteria can and have been shown to escape their initial oral habitat and move to remote sites to exacerbate diseases at sites distant from the oral cavity [35,36]. While we first thought of specific bacteria as the etiological agents of dental diseases, our thinking based on well-designed longitudinal clinical studies has changed our focus to reflect the fact that the consortia of oral bacteria are most likely the provocateurs [29,37]. These consortia operate as a well-organized dysbiotic community and challenge the host’s ability to defend itself [38,39].

(C) Influence of WWCC decision on LAgP publications.

The WWCC’s decision to ignore LAgP as a unique diagnosis has had an outsized influence on diagnosis, treatment, prevention, and scholarship in the areas of microbiology, immunology, and pathobiology of periodontal disease. It was our expectation that the elimination of LAgP as a disease would lead to less research, more limited treatment options, and risk further divorcing our field’s biological initiatives from that of modern medicine. To explore our concern that the new classification has already had an impact on research and clinical scholarship, we performed a database search using mesh terms that included Periodontitis, Aggressive Periodontitis, Localized Aggressive Periodontitis, and Stage III Grade C Periodontitis. We limited the time span of the search to 12 years (6 years prior to the change in terminology, from 2012 to 2017, and 6 years following the classification change from 2018 to 2023). We used these terms to search PubMed, Web of Science, and Scopus databases. Data derived from these searches were used to compare the number of papers published within these time frames in each of the categories specified (Table 1). The results show an overall increase in papers with “Periodontitis” in their title and/or abstract six years following the WWCC but a 33% decrease in those with “Aggressive Periodontitis”. It is abundantly clear that the elimination of LAgP as a case definition has had a major negative impact on the number of publications focusing on patients who previously were classified as LAgP (see Table 1).

## 3. Benefits of Precise Disease Definitions

In the section below, we describe two examples of how well-defined disease definitions lead to accurate diagnosis and improved treatment outcomes. One example relates to dental disease, and one relates to diseases that are sexually transmitted. First, Pediatric Dentistry has deemed it appropriate and beneficial to go with the “splitters” approach and name diseases that have similar outcomes but have distinctive characteristics that can be recognized by clinicians and can be treated in ways that benefit patients [40]. For example, the biological processes of caries, despite their various locations in the mouth, are very similar, and all varieties are related to acid-producing, aciduric microbes that affiliate themselves with tooth surfaces and demineralize the surface and subsurface to create a carious lesion [41]. Nevertheless, for Pediatric dentists, the caries process has been recognized as having distinct subtypes: (1) occlusal caries, (2) proximal caries, and (3) root caries [42]. Moreover, early childhood caries (ECC) has been recognized as a distinct clinical entity [43]. These well-defined diagnoses have led to specific treatments such as (1) sealants used to obliterate occlusal pits and fissures [44], (2) fluoride varnishes for proximal decay [45], and (3) dietary counseling for prevention of ECC [46]. These treatments have resulted in changes in caries prevalence that benefit dental and overall health [47]. Unfortunately, our field’s new WWCC guidelines fail to make similar distinctions for a major subtype of periodontitis, and it is our premise that these guidelines are going to have a negative impact on the treatment and prevention of this unique disease.

Sexually transmitted diseases (STDs) provide a second example where sub-division has had a prominent effect on disease diagnosis treatment success [48]. Naming the specificity of these diseases has led to successful outcomes. For instance, Syphilis is initiated by *Treponema pallidum*, Gonorrhea is due to *Neisseria gonorrhoeae*, and Chlamydia is due to infection by *Chlamydia trachomatis*. Hepatitis B, Herpes Simplex virus (HSV), Human Papillomavirus (HPV), and AIDS initiated by HIV are infections due to specific viruses [49]. In contrast, some diseases can be initiated by a variety of infectious agents and promote morbidity and mortality resulting from disparate confounding factors. For example, Kaposi’s sarcoma can be caused by (1) Herpes virus (HHV-8), (2) AIDS-related HIV, (3) iatrogenic transplant associations, (4) advanced age, most common in Eastern Europeans, Middle Eastern, and Mediterranean men, and (5) endemic Kaposi’s sarcoma, occurring in young people in Africa [50]. These distinctions in diseases and the resulting tissue damage point to distinct therapies since they are complex and are best described by the Damage Response Framework introduced by Casadevall and Pirofski [51,52]. These authors described how HIV, the primary infectious agent in AIDS, typically is not the cause of mortality or morbidity [51,52]. The disease and irreversible tissue and organ damage were due to the immunocompromised host being unable to counteract damage inflicted by otherwise harmless microorganisms [53,54,55]. The lead WWCC authors’ effort to compartmentalize Periodontal Diseases and Conditions fails to distinguish between diagnosis, treatment, and prognosis. On the positive side, their decisions may have improved opportunities for reimbursement by third-party payers, but that outcome remains to be seen.

## 4. Brief History of Landmark Experiments That Support Microbiological Associations with Dental Diseases

(A) Seminal Discoveries of Microbes and Dental Diseases.

Infectious diseases, by definition, have a microbial etiology, and thus, specific infectious agents are used to define the disease of interest. The recognition of microbiological aspects of dental disease began in full force in the earliest days of microbiology, when it first became an accepted field of biology. W.D. Miller, one of the first graduates of the Pennsylvania Dental College (graduating in 1879), went to Berlin to study in the laboratory of Robert Koch. Miller developed the chemo-parasitic theory of caries, suggesting that oral bacteria fermented sugars and produced acids that demineralized enamel [56]. He also developed the focal theory of infection, which suggested that bacteria or products of bacteria can travel to sites distant from the oral cavity and play a role in the development of various diseases that can affect the brain, lungs, and stomach. Sometime thereafter, Kritchevsky and Seguin [57], two French microbiologists, focused on periodontal disease and its treatment. They also believed that oral disease could be implicated in systemic diseases. Over time, the focal theory of infection was abandoned because treatments such as full mouth extraction designed to eliminate oral infection had no impact on the systemic diseases that these oral microbes were proposed to cause. Immunological research in the same era examined the role of oral microbes on immune responsiveness. Notable among them were papers by Beckwith and colleagues, who showed in dramatic fashion how oral bacterial plaque provoked a severe full-body immunological/inflammatory response [58].

However, the emphasis on microbiology and immunology did not receive substantial attention from the dental and research community until two dramatic experiments made a profound and lasting impact. In retrospect, these advances were not without their flaws, and thus, these landmark experiments need to be put into context based on our current understanding of disease [42].

**Figure 1:**
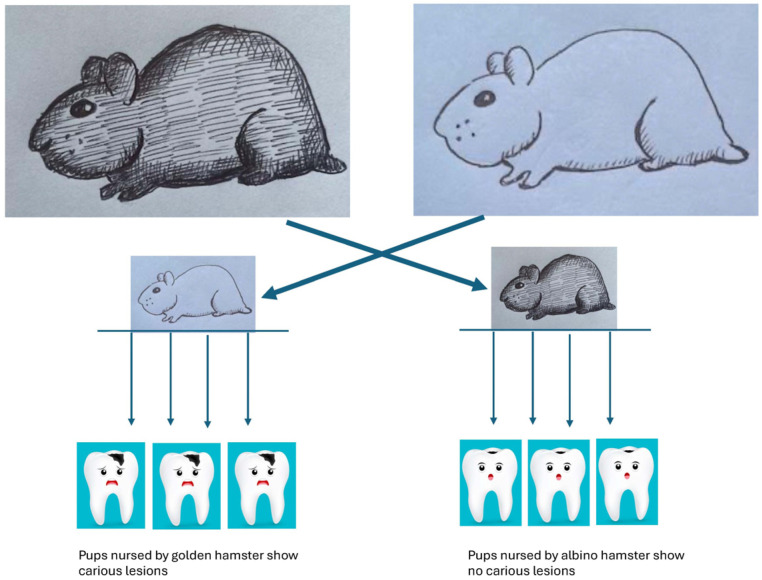
Illustration of the Golden and Albino Hamster experiments showing transmission from mother to child. Golden Hamster Dame (caries positive) is on the left, and Albino Hamster Dame (caries negative) is on the right side. Hamster pups are delivered in a sterile chamber by cesarian section under sterile conditions, and then albino pups are put in the cage of the Golden Hamster pups to be suckled by the Albino Dame (bottom right side of illustration) while the albino pups are suckled by the Golden Hamster Dame (bottom left side of the illustration). As shown, the Golden pups have no caries now (**bottom right**), while the albino pups have caries (**bottom left**).

The series of rigorous experiments by Paul Keyes and colleagues clearly showed that caries could be passed from mother to child when he compared the caries experiences of Golden Hamsters to Albino Hamsters [59]. Keyes was interested in dietary influences on the caries process but accidentally stumbled on the fact that Golden Hamsters were highly susceptible to carious dental lesions while Albino Hamsters were carie-free. By shifting newly born Albino pups into cages with Golden Hamster Dames, he found that these pups, delivered by cesarian section in a germ-free chamber, would now show caries because they were suckled by caries-exposed Dames. In contrast, Albino Dames, who suckled Golden Hamster pups, also delivered by cesarian section, showed no caries. It was found that penicillin could impede the carious process, and finally, it was shown that the agent provocateur was a Gram-positive coccus (later identified as Streptococcus). These experiments showed that caries were caused by oral microbes in animals fed carbohydrates and that the microbes could be passed from mother to child. These experiments were dramatic and demonstrated the importance of acid production and demineralization, but it is important to point out that the experiments were conducted in the absence of a fully developed microbiome. As a result, dysbiosis and homeostatic imbalance were not studied (Figure 1).

**Figure 2:**
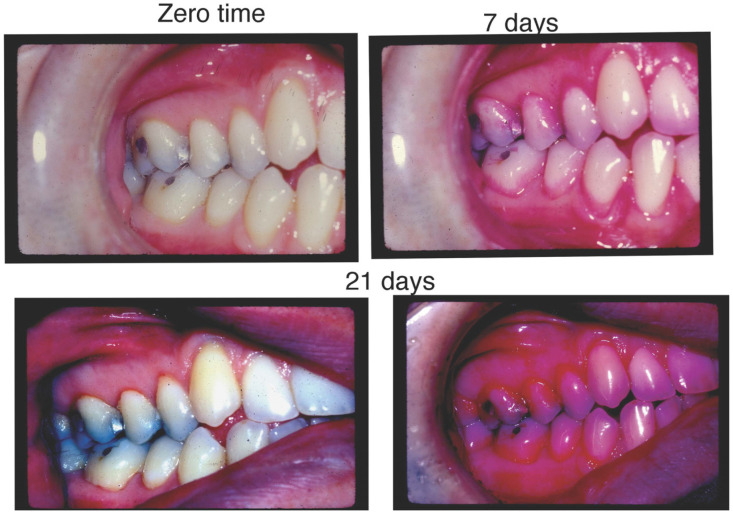
Illustration of the experimental gingivitis model. **Panel Upper Left:** illustration of pre-experimental gingival health of a student prior to abstaining from oral hygiene. **Panel Lower Left:** Gingival indices indicate punctate areas of redness around marginal ginigivae, especially in the upper premolar and molar areas 21 days after abstaining, while incisors show minimal inflammation. **Panel Upper Right:** Plaque disclosure seven days following abstaining from oral hygiene using erythrocin staining. Note minimal levels of plaque around the gingival margin of the teeth. **Panel Lower Right:** Plaque disclosure 21 days after abstaining from oral hygiene. Note the increased intensity of erythrocin staining, which illustrates increased plaque thickness, and how upper anterior teeth show less staining, i.e., less plaque accumulation.

The second seminal experiment was initiated and developed by Harald Löe and colleagues in Denmark [60]. The battleground was the junctional epithelium, the epithelial barrier that formed a boundary between the subgingival pocket area and the gingival and periodontal complex [61]. This complex boundary tissue contained all the elements that could thwart the inward progress and extension of supragingival bacteria to the subgingival space and provoke the ingress of destructive bacterial elements such as toxins, enzymes, antigens, lipoproteins, polysaccharides, and teichoic acids, etc. It was proposed that these substances could weaken the barrier and lead to the ingress of noxious elements, which could result in overall aberrant host responsiveness, tissue alteration, and disease at the local tissue site [62,63]. These experiments opened the door to our recognition that microbes formed on teeth in an ordered, deliberate manner. Exploration of this model showed that over time, the shift from a clean tooth surface to one colonized sequentially by microbial pioneers, followed by a complex mass of bacteria over time, moved from above to below the gum line. This progression from an aerobic to an anaerobic microbiome led to tissue inflammation (Figure 2). These studies showed that resumption of toothbrushing and other oral hygiene methods after abstention for a three-week period caused the removal of microbial masses and a return to gingival health and implied that the supragingival microflora was needed to supply nutrients to subgingival colonizers. This simple, elegant model permitted our colleagues to investigate plaque biofilm development and the associated effect of microbial dysbiosis on tissue inflammation. Sophisticated microbiological and histopathological studies provided a new and vital understanding of how microbes interacted and how they may have affected the underlying epithelial and connective tissue response [64,65]. However, these experiments also illustrated how host inflammation provided a feedback loop for nutritional supplementation that might have accounted for microbial expansion [66]. It was also shown that not all subjects responded in the same way and to the same extent relative to the microbiological challenge and that within the same mouth, different sites responded differently [67,68]. While now appreciated to be much more complicated than previously thought, these experiments resulted in more questions than answers and stimulated new areas of research that have moved well beyond dentistry. The whole field of coaggregation initiated by Paul Kolenbrander and colleagues was derived from these primitive but elegant experiments [69,70].

These understandings re-awakened interest in the movement of bacteria from oral to distant sites [63]. Local destruction was thought to result from the direct result of bacterial by-products such as enzymes, toxins, etc., or from an overactive host response initially designed to stem the tide in favor of healing [71]. However, many believed that an excessive host response could also be responsible for local tissue destruction and/or disease. The experimental gingivitis model of Harald Löe and colleagues provided a close examination of the chronological association of oral bacteria in supragingival plaque and the coordinated dysregulation of the host response to accumulating bacteria [72] (Figure 2). In the figure above, a dental student abstained from all oral hygiene for a 3-week period, and both gingival and plaque indices were recorded at 7, 14, and 21 days after abstention. In addition, after this 21-day period, the student brushed and flossed their teeth, and the plaque and its resulting gingivitis returned to pre-abstention levels. The figure above illustrates the dramatic changes resulting from abstinence from oral hygiene (Figure 2).

(B) Microbiology/*A. actinomycetemcomitans* and LAgP.

In an effort to abbreviate the long history of aggressive periodontitis in adolescents, it is worth mentioning the significance of the original recognition of what was first called periodontosis by Dr. Bernhard Gottlieb in Vienna in 1923 [10]. In the “modern era”, Dr. Paul Baer (1971) presented a prescient, more detailed clinical description of this entity, then called Localized Juvenile Periodontitis (LJP) [6]. His description depicted rapid bone loss in younger individuals and was founded on a minimum of the following five clinical features: (1) the age of onset, (2) a family history, (3) the lack of relationship between local factors and deep pockets, (4) the rapid rate of progression, and (5) the effect on primary teeth. In the over five decades since this publication, the name of this disease and the disease itself have often been debated [1,3,73]. Unfortunately, since Baer’s descriptive and thorough paper, many twists and turns have resulted in great confusion that has and will continue to seriously impede efforts to understand the microbiology, pathology, clinical definition, and treatment of this silent (often painless symptomatology) and uncommon condition.

In 1976, *Actinobacillus actinomycetemcomitans* (*Aa*; now *Aggregatibacter*) was shown to be associated with what was then called Localized Juvenile Periodontitis (LJP) [74,75]. This discovery coincided with an effort to demonstrate that specific microbial entities were responsible for specific forms of periodontal disease and led to the birth of the Specific Plaque Hypothesis (SPH) as its counter-part the Non-Specific Plaque Hypothesis (NSPH) [76,77]. *Aa*, a Gram-negative capnophilic microbe, was discovered in 1912 by Klinger [78] in a disease called Actinomycosis (lumpy jaw disease). Interestingly, *Aa* was not presented as the sole actor in this disease, and *Aa* was shown to have acted in concert with *Actinomyces israelii*; hence, the species name “actinomycetemcomitans” or in common with Actinomyces [78]. The SPH stimulated a significant body of research that explored specific microbes and their relationship to specific clinically recognizable forms of disease [79]. They were significant hypotheses for their time and stimulated a voluminous literature that examined the biological features of several microbes associated with both caries and periodontitis [80,81,82]. This intellectually challenging approach added significant scientific information to microbial features related to these diseases that were here-to-fore unexplored [83]. The understanding of dental diseases, as well as the biology and pathogenicity of several microorganisms associated with distinct forms of dental disease studied, benefited from these broad hypotheses. These microbes included but were not limited to *Streptococcus mutans* and its relationship to caries [41], *Actinobacillus* (now *Aggregatibacter*) *actinomycetemcomitans* and its relationship to LJP [74,75], *Bacteroides melaninogenicus* (now *Porphyromonas gingivalis*) [81,84] and its relationship to adult periodontitis, and *Actinomyces viscosus* and its relationship to root caries [85,86]. In retrospect, this appears to be a naïve approach, but nevertheless, it stimulated a plethora of research that led to a better understanding of glucans and dextrans in the case of *S. mutans* [87], leukotoxin and cytolethal distending toxin in the case of *Aa* [88], gingipains [89], hemolysins [90], and collagenases in the case of *P. gingivalis*, etc. [91]. The choice between the SPH and the NSPH was replaced by the Ecological Plaque Hypothesis [92], which, though modified over the years, has highlighted how dental diseases are influenced by ecological interactions and how ecological interactions can influence and modify dental diseases.

(C) Bradford–Hill Guidelines for Determining Causation: Association of *A. actinomycetemcomitans* in a Microbial Consortium with LAgP.

Of all dental diseases, LAgP is the closest to fulfilling the Bradford–Hill Guidelines for the association of provocative agents and disease [93]. LAgP, if defined in the most stringent manner, focusing on (1) adolescents with bone loss in the molar or incisor region and (2) young individuals typically of African descent, satisfies six of the nine aspects of the Bradford–Hill criteria. These associations are required to show (1) temporality, or exposure to the agent prior to disease; (2) strength of association, or the level of association of the microbe as determined statistically; (3) a biological gradient or dose-related response of a biologically-active substance as related to tissue damage, thus, the higher the exposure, the greater the disease; (4) consistency or reproducibility of critical experiments by others; (5) plausibility of the association of the agent with its pathological consequences such as tissue damage caused by the microbe; (6) alteration of the disease, or experimental evidence that intervention alters the provoking agent (either the bacteria, bacterial complex, or virulence agents) and the disease (See Table 2).

The associations presented in items 1 to 6 in the table below are based on a longitudinal model designed to study the transition from health to disease and demonstrate that a specified consortium of microbes, in addition to *Aa*, is required for the disease to occur (*Aa* is necessary but not sufficient) [29]. It is possible that the consortia can be expanded to include other microbes, but the original observation and the replication of the consortia by others who conducted longitudinal studies in well-defined adolescent patients of African descent have shown that *Aa* is a critical member in the disease process (see Table 2). We suggest that *Aa* is a social influencer that, despite its low level, has an outsized influence on microbial community geography and behavioral interactions. The most likely rationale is that *Aa* impairs local immune responsiveness by production of leukotoxin, cytolethal distending toxin, and upregulation of complement resistance to allow for its survival as well as the survival of less adaptable subgingival microbes. Models and clinical studies suggest that the consortia consist of *Aa* and *Filifactor alocis*, among other subgingival microbes [29,94]. It appears as if *F. alocis* invades the biofilm at the later stages of subgingival biofilm formation but then provokes an aggressive and damaging host response by continuing to alter the local immune responsiveness with the production of its own form of leukotoxin. It appears as if both *Aa* and *F. alocis* relationships with disease could be strain related [95]. Other studies have found somewhat different consortia associated with periodontitis in older patients [96]. There is a great deal of evidence that points to bacteria as initiators of cell-mediated tissue loss in periodontitis [80,81,97]. Linking this to typical environmental factors of suspicion is still in its infancy. In contrast, studies of carious lesions are clearly dependent on environmental factors, such as carbohydrate consumption, that stimulate the growth and survival of acid-producing–acid-loving microbes [41,83]. It is still too early to conclude, but highly likely, that nutritional elements derived from the host and bacterial community co-inhabitants are important in microbial biogeography in LAgP, but these factors require more in-depth study, another reason to maintain recognition of the condition as a distinct clinical diagnosis.

**Table 2:** Causation of disease by microbial consortia assessed by Bradford–Hill criteria.

Hill Criteria	Example	Feasibility Yes/No?	Impact of Study and Reference
1. Temporal relationship	Exposure to agent precedes outcome	Yes	Longitudinal; healthy controls; age approp *Aa*; [98,99] Longitudinal; health controls age approp *Aa* + consort; [29,100]
2. Strength of Association	Size of association determined statistically	Yes	Show stats *Aa*; [98] Show stats *Aa* + consort; [29,100]
3. Dose-Response	^exposure > ^response	Yes	Measure consort vs. *Aa* alone; [29,100]
4. Consistency	Experiments reproduced	Yes	Show consort X-sect; [101,102] Show consort longitude; [29,100]
5. Plausibility	Assoc agrees with pathobiological explanations	Yes	Cdt has impact; [103] Ltx has impact; [95] Consortia passed from mother with disease to Child: local debridement improves inflammation, but consort remains; [104]. Consort metabolomics; [94]
6. Experimental evidence	Disease altered By intervention	Yes	Tetracycline admin reduces disease; [105] Tetracycline eliminates *Aa* and reduces disease; [106] Amox/Metra reduces disease, no antibiotic, no improvement; [107]
7. Alternative explanation	Rule out other explanations	? Open ? Open	[29,98,100]
8. Specificity	Cause produces effect	Yes	Flp and no disease; [108] Ltx and more bone loss; [109] Pga B is modified, and disease is reduced; [110]
9. Coherence	Theory consistent with Existing knowledge	Yes	Ltx and infections; [111] Cdt and infections; [112] Metabolomics and consortia; [94]

To support these associations in the Bradford–Hill guidelines, we required a minimum of three independent longitudinal studies that test for categories 1 through 6. For positive proof, at least two to three longitudinal studies were required to show a microbial consortia or virulence complex that was implicated in the disease prior to detection of disease. Due to the dearth of longitudinal studies performed using the appropriate populations, we proposed that one of the three studies showing that the presence of *Aa* alone could, at this moment, satisfy categories 1 through 6. Guidelines 7 to 9 were also assessed. Guideline 7 evaluates specificity and requires that the study demonstrates that a cause can produce an effect. Guideline 8 evaluates experimental alteration, which requires demonstrating that the disease can be altered by an intervention that reduces the overall damage response. Guideline 9 discusses coherence, which requires that the theory presented is in keeping with existing knowledge. Guidelines 7 to 9 have been limited to *Aa* and its effects because studies with the consortia in these categories have not yet been reported. While newer approaches to data integration have been used to expand the interpretation of the Bradford–Hill guidelines, they remain an important way to associate infectious agents with the etiology of disease.

## 5. Damage/Response Framework and LAgP

(A) Overview.

**Figure 3:**
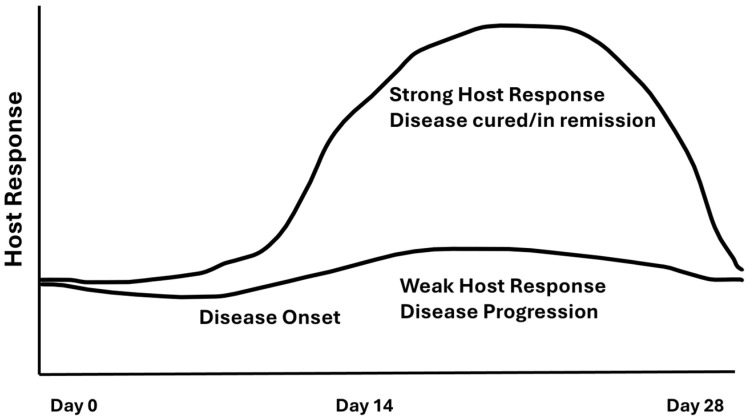
Diagram of Damage/Response. The solid upper line represents a normal healthy host response. At day zero an infection occurs driven by either a bacterial or viral challenge to the host. Following the microbial challenge after some delay the disease develops and the host response occurs. Disease resolves and the host response tapers to avoid furter tissue damage. The bolded straight line on the bottom represents an inadequate host response. Here tissue damage continues until an adequate host response occurs. Thus the bolded bottom line represents a muted host response and continued tissue damage.

During the early period of microbiological studies, there was also an appropriate and important emphasis placed on the host immunological response [113]. At this time, the disease process was thought of as a war between “bad” bacteria and the host’s responsiveness to those bacteria [114]. Notable experiments and descriptions by Page and Schroeder [64], work by Taubman et al. [115], Genco and Sanz [116], Taichman and colleagues [117,118], and Lehner and colleagues [113] emphasized stages in the inflammatory process that highlighted the prominence of specific cells such as polymorphonuclear leukocytes (PMNs), monocytes, macrophages, and plasma cells. The importance of each of these cell types in the process of tissue damage was carefully described, and particular emphasis was placed on plasma cells and their ability to destroy bone via osteoclastic activity. In parallel to this work, hemolysins, collagenases, and a host of destructive microbial factors derived from *B. melaninogenicus*, as well as a leukotoxin derived from *Aa*, were uncovered. The apparent emphasis and conflicts between microbiologists and immunologists became perceptible in many forms of infectious diseases [114]. More recently, periodontal research has shown that shifting levels of cytokines and chemokines act as signaling molecules that encourage cellular activity [68]. The emergence of the Damage/Response Framework shifted the emphasis away from a competition between microbes and the host to the intimate interaction between the microbe and its host in relation to disease progression [51] (Figure 3).

The oral cavity provides an ideal place to study host/microbial interactions [119,120]. First and foremost, the oral cavity is the entry point for most external substances. Material derived from the mouth is easily accessible for longitudinal analysis. Analysis of the interaction of cellular and acellular material can be collected and normalized for quantitative assessment. Oral collections can be performed painlessly, without aggressive/invasive/destructive methodologies, and with little to no interference with bodily function [119]. Finally, host influences, as well as external influences such as diet, radiation, circadian rhythms, drug effects, stress, aging, trauma healing, etc., can be documented in vivo [121,122].

The landmark experiments by Keyes and Löe provided the impetus for studies of microbial colonization of teeth and oral soft tissue from birth to senescence, which proved to be thorough and accurate and more easily studied than microbial colonization of the gut lining [123]. While feces collection is a way of studying gut colonization, microbial contact with gut epithelium can only be studied using invasive, colonoscopic methods [124]. The experimental model developed by Löe and colleagues has led to a more complete understanding of biofilm formation, coaggregation, and host responsiveness to microbial challenges [125]. Most recently, this model has been amplified to examine chemokine and cytokine levels over the time course of the 3-week non-brushing model [68]. Unfortunately, many assumptions made about the host response to the immediate bacterial challenge in this model have only recently been documented. It is quite conceivable that microbial factors move through the subgingival epithelial barrier formed by the junctional and sulcular epithelium and that local host inflammation plays an important role in microbial dysbiosis [62,126].

(B) Damage.

i. Up until the mid-1980s, diagnostic microbiology, immunization, and antibiotic therapy have proven to provide a crucially important strategy used to control many prevalent infections caused by identifiable microorganisms [127]. However, since infections such as acquired immunodeficiency syndrome (AIDS), new insights into complex diseases that result from host modification leading to lethal progression due to secondary well-defined infections have taken center stage. These complex secondary infections required a holistic approach to diagnosis, prevention, and treatment of disease, hence the evolution of the damage/response framework [51].

Data acquired over the last 60 years of research in microbiology and immunology suggest that LAgP, in several ways, shows similarity to a local form of AIDS [29]. This realization contrasts with typical blood-borne mono-infections such as Syphilis (*Treponema pallidum*), Diphtheria (*Corynebacterium diphtheriae*), or Anthrax (*Bacillus anthracis*) or any blood-borne mono-infection that spreads disease-producing toxins [128]. In the localized immunomodulated AIDS-type scenario (LAgP/type scenario), once the barrier effect at the local site has been affected, bacteria from subgingival plaque weaken the local epithelial barrier and can enter the connective tissue, alter it, and then invade the bloodstream and translocate to distant sites [129,130].

In the context of this newly emerging concept of complex/host compromised/multi-species/multi-layered infection, the oral cavity provides an ideal environment to study diagnosis, initiation, and progression of diseases that involve multi-species microbial interactions and that produce altered host responses that fail to control shifting microbial challenges [119]. These sentiments are not meant to minimize post-infection host healing efforts to repair damage caused by infectious insults. On the contrary, because of access and exposure, the oral cavity can provide an ideal environment for studying disease initiation, metabolic processes, and disease progression in these complex multi-layered infections [131].

ii. *A. actinomycetemcomitans* is a pathobiont that is opportunistic in its own regard, but *Aa* also creates a perturbation of homeostasis by modulating PMNs, macrophages, lymphocytes, and other local immune functions [9,132,133,134]. Periodontitis, particularly LAgP, can be described as a disease that forms an acquired immunomodulated host response at the local level [135]. *Aa* and other microbes can be associated with disease susceptibility created by an imbalance between a dysbiotic microbiome, resulting in a perturbed host homeostasis [79]. The damage process can be further altered by subsets of other pathobionts, such as *P. gingivalis* [37] and *F. alocis* [136], such that exacerbated local damage can occur as a result of the overwhelming challenge due to the overgrowth of otherwise commensal/opportunistic microbes (*F. nucleatum*, *S. parasanguinis*) [29,94]. Overgrowth of these specific pathobionts and commensals in an otherwise compromised local immune response can now diminish the reparative capacity at the local site and enhance the resulting tissue damage [137]. The damage can remain localized, but in certain cases, an altered barrier can result in the movement of pathobionts and/or commensals to sites distant from the oral cavity [138]. Several experiments have replicated the early work of Okell and Elliott [129] showing transient bacteremias emanating from oral procedures can move many oral microbes to distant sites through the bloodstream [130,139]. Mechanical dental procedures such as scaling and root planning, as well as flossing, brushing, and eating an apple, can also induce transient bacteremias [140,141].

(C) Response.

i. Host: What we lack in this area of study is sufficient time course experiments that document host cellular changes that evolve from health to early, middle, and later stages of periodontal disease development [64]. Studies of these events have occurred more directly in experiments on endodontic lesions [142]. While different in some respects, oral biologists should be encouraged to examine the similarities and differences in periodontal and endodontic lesions. Just as the experimental gingivitis model enlightened our understanding of time-related events associated with microbial development in both the supragingival and subgingival environment, we still need to decipher the passage of substances from the “pocket” to and through the epithelial basement membrane to challenge the immediate area below the barrier membrane. It has taken 60 years from the inception of this classical gingivitis model to document interbacterial signaling distances and bacterial by-product host–cell interactions. However, we have now come to a time when technological advances have caught up to our theoretical understandings. We are now on the threshold of the merging of ideas and technological advances. DNA techniques [143], bar-coding [144], and CLASI_FISH [145] technologies now provide us with the tools required to study subgingival bacterial biogeography and cellular phenotypic responsiveness in a time-related manner [146]. In this quest for a more complete understanding of microbial–host interactions, we, as oral biologists, can now study these events in a sequential manner with easy access to microbial- and host-induced inflammatory response elements [39].

To re-iterate, early plaque development and initiation of gingivitis provided a straightforward path. Plaque could be collected easily, for example, from a tooth surrogate, which, when placed in the mouth, could serve as an aseptic surface for microbial associations over time [147,148,149]. Microbiological, immunological, and DNA technologies are now being used to catalog biogeography. A tooth analog can be placed at or just below the gum line to document the transition from supra to subgingival biofilm formation [150]. Documentation of subgingival plaque and the host response to supragingival plaque is more difficult. Early efforts to use mylar strips, cemental strips, and polyvinyl strips have yielded useful but incomplete information [147,148]. There have been several reports that have tried to document the complexity of subgingival plaque, but since this is the focal point for the spread of commensal oral microbes throughout the body, more must be done.

ii. Clinical Measurements as Determinants of Treatment Failure or Success. In the overall scheme of things, we have determined through both clinical observations and scientific testing that microbial plaque leads to gingival inflammation, which leads to tissue destruction, barrier alterations, pocketing, attachment loss, and then bone loss [151]. While this sequence of events has been observed in humans and replicated in animals, the timing of these events and their route of progression can be influenced and altered by a shifting microbiota as well as host responsiveness [63]. In the presence of sophisticated biological methodologies, our clinical measurement methods have remained stagnant and reliant on a periodontal probe and an X-ray.

It is hard to imagine that periodontitis is not due to a dysbiotic microbiome, which results in a disease defined by tissue destruction and weakened tooth support [151]. In most, if not all, diseases, successful treatment is typically measured by repair of altered tissue and/or alternatively in reduced recurrence or relapse of disease [30]. In most cases, treatment of periodontitis has relied on tissue repair as demonstrated by pocket depth reduction or attachment level gain [31]. However, in recent years, many studies have shown that oral microbes can travel through the bloodstream, and these oral bacteria can exacerbate systemic diseases at distant sites, such as colorectal cancer, heart disease, etc. [152]. In these cases, it might be prudent to look at treatment success in an alternative manner, such as the prevention of disease progression, recurrence, or relapse, as a way of reducing initiation of diseases at distant sites.

## 6. Distinct Characteristics of LAgP and an Alternative Approach for Assessing Treatment Success

(A) Distinct Characteristics of LAgP: An Illustrative Case.

This case is presented to illustrate the distinctive nature of aggressive periodontal bone loss in a 20-year-old patient who reported to our clinic (RSDM) with extensive periodontal disease. After obtaining consent (IRB:PRO#012008035; year = 2009), we collected subgingival plaque, saliva, and buccal cells for analysis. The subgingival sample taken from various healthy and diseased sites had the “b” serotype of *Aa* with the JP2 promoter region. The patient had only one strain of *Aa* in his subgingival microbiome isolated from both healthy and diseased sites, with substantially more *Aa* isolated from diseased sites. We tested the *Aa* isolates for antibiotic sensitivity and for the presence of the hbpA-1, hpbA-2, and tbp-A pseudogenes using primers reported by Haubek et al. [153]. Saliva was assessed for salivary anti-*S. mutans* activity and buccal cells were used for the detection of the lactoferrin (LF) single nucleotide polymorphism associated with anti-*S. mutans* activity [154]. Our primary goal was to use our laboratory data to provide information about *Aa* antibiotic sensitivity, which could act as a supplement to treatment aimed at resolving this progressive disease in this young patient. The focus on *Aa* was pragmatic because our previous data had suggested that *Aa* was necessary but subsequently proved to be insufficient on its own to cause disease [29]; conversely, assessment of the complete consortia was impractical at that time. However, several of our other laboratory assessments proved useful.

**Figure 4:**
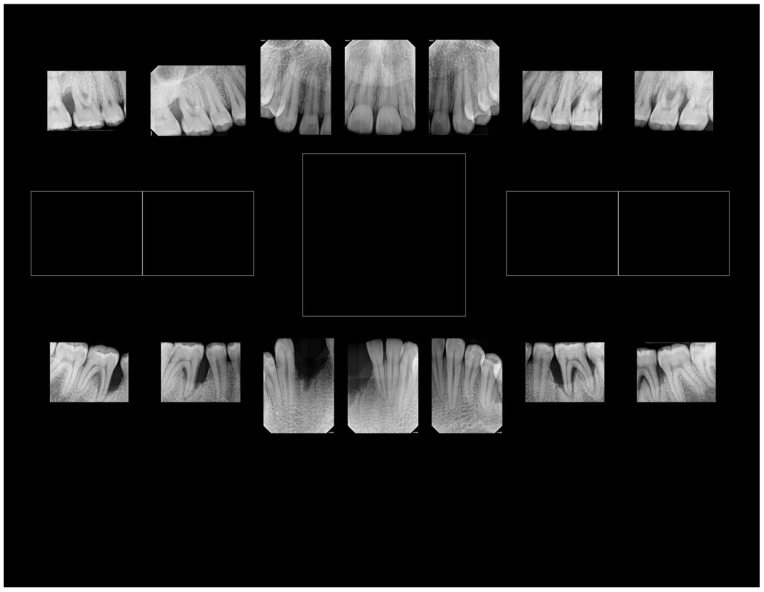
Radiographs of a patient with significant pocket depth and bone loss. Shows loss of two mandibular incisors and extensive bone loss in the first molar region,. Note the lack of carious lesions on radiographs throughout the dentition.

While we recovered *Aa* from the diseased site, we cannot attribute disease to the presence of *Aa*. Microbial causation can only be implied if the bacterium preceded disease at the site of disease initiation [41]. Therefore, in this case, linking *Aa* to disease initiation and development can only be seen as speculative. Second, based on the complex patient history, it is reasonable to conclude that confounding social, psychological, and ecological modifiers could have contributed to a diminished host response, factors that could clearly be implicated in disease progression [42]. Based on antibiotic sensitivity testing, we ruled out the use of penicillin derivatives due to *Aa* insensitivity, which was a clinically useful finding. Furthermore, the age of the patient, the tooth loss attributed to periodontal disease, and the extent of bone loss indicated an aggressive nature of localized disease in this patient (Figure 4). We posit, based on tooth location and the patient’s history, that the loss of two mandibular incisors was due to extensive periodontal disease (Figure 4). This conclusion appears to be a realistic appraisal of tooth loss as a consequence of (1) the dramatic level of bone loss in the existing molars and (2) the complete lack of caries in this subject’s mouth (and the fact that the mandibular incisors lost are not typically vulnerable to caries) [155]. Testing for salivary anti-S. *mutans* activity gave an incomplete picture since several other oral microorganisms can also be related to caries [156]. The fact that anti-LF antibodies had no effect on *S. mutans* suggests that factors other than LF were responsible for the anti-*S. mutans* activity. This finding agrees with our previous data, where 20–30% of subjects tested showed factors independent of LF that also killed *S. mutans* [154]. The level of bone loss and lack of proximal decay reflects a pattern seen in many cases of LAgP [157,158]. Finally, point mutations in the hpbA-1 and tnp-B pseudogenes suggested that the patient was of West African descent [153].

**Figure 5:**
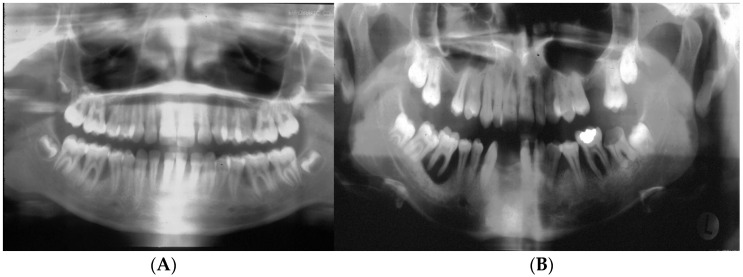
Panoramic radiographs of cases of aggressive periodontitis in adolescents. Panel (**A**) shows a panoramic view of excessive bone loss in the first molars and no carious lesions. Panel (**B**) shows more extensive disease in an adolescent with bone loss around the molars and missing molars and incisors with occlusal, but proximal decay is related to a blow-out occlusal lesion in the mandibular right second molar.

In summary, the clinical presentation, coupled with the presence of minimal plaque, the absence of proximal decay, severe periodontal disease, and the presence of *Aa*, all present a strong argument that LAgP is a disease uniquely distinguishable from periodontitis that occurs in adults (Figure 5).

(B) Another way of Assessing Periodontitis Treatment Success.

Many of our field’s most revered longitudinal studies have relied on averaging pocket depth reduction or attachment level gain resulting from a specific treatment. Oftentimes, data are presented as a reduction of probing pocket depth or attachment gain over a 3-month to 1-year period after completion of treatment [31]. These measurements are usually averaged over 28 teeth, each tooth having six measured surfaces, thus yielding up to a total of 168 probable or measurable sites per mouth. To illustrate the issues related to averaging an overall dental response, we compared Cases A and B. Case A represents a patient that has two probable pockets, with each site having a 10 mm probing pocket depth on initial examination. Thus, we start with two 10 mm pockets on the distomesial and lingual–mesial surfaces of the left and right mandibular first molar or a total of 40 mm of pocketing at four diseased sites (4 × 10 = 40). The remaining 164 probable sites have pockets of 3 mm. Cumulatively, these sites have a total of 492 mm (164 sites × 3 mm pockets per site = 492 mm of total probing depth). Overall, the patient presents with a total of 532 mm of pocketing in 168 probable sites (492 + 40 = 532 mm) over the whole mouth or an average of 3.17 mm of pocketing per site. After treatment, consisting of deep scaling and root planning, the four 10 mm pockets were reduced to 8 mm (now a total of 32 mm), while the remaining 164 sites remain at 3 mm (164 × 3 = 492). After treatment, the total pocketing in the mouth is 492 mm + 32 mm = 524 mm. In this scenario, the average pocket depth is 524/168 = 3.12 mm per site, a reduction of only 0.05 mm (3.17 mm to 3.12 mm). In case B pockets are reduced from 10 mm to 2 mm and thus pocket depth scores go from 3.17 to 2.97 (492 + 2 mm × 4 = 8 mm or a total of 500 mm; 500/168 = 2.97 mm). Thus in Case B a reduction of 20 mm is seen as compared to one of 0.05 mm. There has been some effort recently to focus on the categorization of changes in pocket depth reduction or attachment level gain in sites of low to moderate to high risk, but this has not been fully conceptualized or actuated [159].

When disease returns at specific sites, it is almost always associated with a dysbiotic microbiome [82,86]. Therefore, in contrast to measuring pocket depth reduction or attachment gain that we are accustomed to doing, a disease re-occurrence reduction or relapse to infection model is proposed. This can be applied independently or combined with standard measurements of improvement at the disease-affected sites. In this relapse model, we question how treatment has reduced the risk of re-occurrence of the disease. This disease re-occurrence reduction model implies that a goal of treatment is to enhance the local tissue barrier effect, such that the spreading of the infective oral bacteria from the site below the gum line to areas distant from the oral cavity is another goal of treatment. For example, in Case A, re-infection and return of deeply infected sites might likely occur 4 weeks post-treatment intervention, whereas in Case B, it might take 2 years or more for re-infection and reemergence of large pocket depths to re-occur. This pocket depth comparison presents an example of how oversight of re-infection in the oral dentition could provide a superior environment for patient overall health. The re-infection model takes into account the fact that while in our dental plaque model, we establish “good guys” and “bad guys” in a supra or subgingival plaque environment, we are fully aware of the fact that some of the so-called “good guys” become “bad guys” when they move past the local epithelial barrier and through the bloodstream such that they can then colonize heart valves, colon cells, lung or brain tissue [160]. Our proposed model moves away from the concept that infection and disease are a war between bacteria and the host and moves toward the concept that disease is the result of a damage/response ratio. The overall result appears to be dependent on coping mechanisms by the immune system that are designed to successfully reduce the consequences and spread of microbial/viral/fungal interactions from the local site to sites distant from their origin [161].

## 7. Conclusions: Future Challenges/Recommendations for the Clinical and Research Community

Consensus meetings in medicine and dentistry usually gather experts in the field with the goal of reviewing the current literature to define disease diagnoses and the most effective treatments [162]. The meetings are intended to generate a report that will be presented to the world of clinical scientists and practitioners with guidelines based on careful evaluation of information currently available. The resulting conclusions can have a profound influence on preventive, diagnostic, and treatment strategies for years into the future. The rules and regulations concerned with the conduct and presentation of material derived from consensus conferences vary widely and sometimes appear to be determined in a haphazard manner [162,163,164]. As such, the WWC organizers’ re-classification of periodontitis case definitions is confounded by the use of overlapping and inaccurate clinical definitions that, in the case of LAgP, disregarded key clinical features that set it apart from other forms of periodontitis [21]. The decision to eliminate LAgP as a distinct form of periodontitis may have been partly because the population most often affected is not seen in some regions of the world.

While there is a great deal of inconsistency in the rules and regulations of consensus conferences, there are several examples that set standards for good practice. One example of a well-performed consensus conference required a vote of 70–80% or higher agreement by the experts involved to reach what could be labeled as a consensus [162,163,164]. Even with the 80% rule, consensus conference participants in the minority were required to publish the basis for their dissenting point of view so that readers could have a clear understanding as to how decisions were made, what the opposing views were, and the future directions projected [165]. This process should be used in the next WWCC.

To avoid problems that occurred in the most recent WWCC, it is recommended that future Consensus Conferences include the following characteristics: (1) A criteria statement made at the outset of the conference requiring that each foundational paper meet specific standards (e.g., use of the Delphi process, use of longitudinal data, clear case definitions, etc.); (2) a statement clearly articulating the percentage of participants that are required to agree with recommendations made by Conference attendees in order for the recommendation to be considered a consensus opinion (i.e., 70–80% agreement); (3) the presentation of dissenting points of view with explanations for such views so that readers of the report can gain an insight into the views of all participants; and (4) a concluding statement which should present realistic suggestions designed to improve case definitions in the future [165].

Finally, based on the data and examples presented in this review, we suggest that re-instating LAgP as a distinct disease entity should be carefully reconsidered. This would not in any manner replace Staging and Grading. However, for the reasons reviewed in this paper, the WWCC system should carefully consider making a distinction between this unique disease and the generally accepted adult form of periodontitis for the betterment of both research and clinical practice. In addition, as a result of the localized nature of this disease, we propose an expansion of treatment goals to include the reduction of re-emergence of new cycles of infection to serve as a supplementary way of assessing treatment success. This point speaks to the concern that re-infection of the local site may lead to the passage of either oral commensals or pathobionts from the local periodontal site to sites distant from the oral cavity. Therefore, the goals of treatment for periodontitis or any other dental infection are both to support local healing and repair coupled with the goal of reducing translocation of oral microbes throughout the body to limit the scope and extent of damage contributing to diseases occurring at sites distant from the oral cavity.

In an effort to engage our academic and clinical community in the process aimed at expanding the classification and therapeutic approaches as they relate to LAgP, we propose the following questions: Will the material we have presented in this review lead to a renewed enthusiasm for the reinstatement of LAgP as a distinct disease entity? (2) Will our community adopt the proposed addition of reduction in relapse of infection as an additional measure of treatment success in unique disease categories? For example, will the use of specifically targeted antibiotics typically used to reverse pocket depth and improve the gain of attachment also delay the time to relapse of infection and, as such, be considered as an additional measure of treatment success? (3) If this expanded assessment of treatment success is adopted, will this limit the potential for local dental infections to exacerbate systemic disease at sites distant from the oral cavity? The goal of this review has been to raise questions that can expand the horizons of dental research, diagnosis, and treatment so that clinicians and researchers can more effectively study how dental infections can be assessed and controlled for the benefit of the overall health and well-being of our patients.

## Figures and Tables

**Table 1:** Numbers of publications by periodontitis disease categories 2012–2023.

Disease Category	Pub Med Years		Web of Science Years		Scopus Years	
	2012–2017	2018–2023	2012–2017	2018–2023	2012–2017	2018–2023
Periodontitis	6492	6287	10,181	16,420	13,000	18,926
Aggressive Periodontitis	884	534	1053	817	1030	679
Localized Aggressive Periodontitis	179	114	134	102	118	80
* Stage III Grade C Periodontitis		* 148		* 138		* 135

Comparisons between 6 years prior and 6 years following category changes are seen below as percentages disclosed by each search engine. Periodontitis changes varied from −3% to +38%. Aggressive Periodontitis changes varied from −22% to −40%. Localized Aggressive Periodontitis changes varied from −24% to −36%. * Stage III Grade C includes all forms of Aggressive Periodontitis and does not discriminate between adolescents and adults who have the disease; thus, the number of publications is at a minimum of 60% less when compared to LAgP; in addition, 20% of these publications define the new classification and did not present clinical data.
